# Educational disparities in cancer incidence, stage, and survival in Oslo

**DOI:** 10.1007/s43999-024-00037-x

**Published:** 2024-01-29

**Authors:** Afaf Al-Rammahy, Elin Anita Fadum, Yngvar Nilssen, Inger Kristin Larsen, Erlend Hem, Berit Horn Bringedal

**Affiliations:** 1 Department of Behavioural Medicine, Institute of Basic Medical Sciences, Faculty of Medicine, University of Oslo, Oslo, Norway; 2 Institute for Studies of the Medical Profession, PO Box 1152, NO-0107 Sentrum, Oslo, Norway; 3 Department of Registration, Cancer Registry of Norway, Oslo, Norway; 4 The Norwegian Armed Forces Joint Medical Services, Institute for Military Epidemiology, Sessvollmoen, Norway

**Keywords:** Norway, Social inequality, Cancer incidence, Cancer survival

## Abstract

**Objectives:**

This study aimed to examine disparities in cancer incidence, stage at diagnosis, and survival rates across districts with differences in education levels in Oslo, Norway.

**Methods:**

Aggregated data from the Cancer Registry of Norway in the period 2013-2021 were used to describe the distribution of cancer incidence and survival across Oslo’s 15 administrative districts, subsequently grouped into three areas based on the population’s level of education. Age-standardised incidence rates and five-year relative survival were calculated for colon, rectal, lung, melanoma, breast, and prostate cancer. The stage at the time of diagnosis was categorised as localised, regional, distant, and unknown for all cancer types except breast cancer, which was categorised into stage I-IV and unknown.

**Results:**

Mid- and high-education areas had higher incidences of breast, melanoma, and prostate cancer, while the low-education area had higher incidence rates for lung cancer. The low-education area had a higher proportion diagnosed at a distant stage than the other groups for all cancer types studied, except breast cancer. The mid- and high-education areas had higher five-year relative survival rates overall.

**Conclusions:**

Incidence, stage at diagnosis, and survival varied between education areas. The variation indicates disparities in healthcare access, quality of care, and health behaviours. Addressing these disparities can help improve overall health outcomes and promote health equity.

**Supplementary Information:**

The online version contains supplementary material available at 10.1007/s43999-024-00037-x.

## Introduction

Norway is often regarded as a leading example of an egalitarian society. Income inequality is among the lowest of the OECD countries [1], and health care is universally covered through the National Insurance Scheme (Folketrygden). Access to specialist health services is regulated by The Act on Patients’ Rights [2] and The Act on Specialist Health Services [3]. The first section of both Acts state that equality in access and quality are overall aims. Further, according to an OECD-evaluation, Norway’s cancer care is ranked better than that of the EU [4].

Still, nationwide studies demonstrate social and geographical disparities in cancer incidence [5, 6]. Other studies have found that patients’ education and place of residence may affect how early cancer is diagnosed, as well as treatment for lung and colorectal cancer [6–9]. Studies also find that cancer patients with higher socioeconomic status have better survival rates [10–13]

Although there are nationwide studies on this topic, we know little about the incidence and survival rates at the regional level. Examining smaller areas where, e.g., travel distance to health services is similar, can provide further insight into the mechanisms behind variations in cancer incidence and survival. This is valuable information for preventive efforts.

In Oslo, Norway, life expectancy varies between the 15 districts, with up to a six-year-difference [14]. Education level serves as a key social determinant of health [15], and may influence access to healthcare, health behaviours and overall health outcomes [16]. Several national studies have found an association between educational level and length of life expectancy [17, 18]. An analysis of life expectancy in Oslo found that the differences were attributed to socio-economic characteristics and behaviours exhibited by the residents. Specifically, men’s life expectancy was lower in districts with a low percentage of individuals with higher education, but other factors, such as the proportion of men living alone or the number of men using tobacco, were also associated with life expectancy differences [19].

In this study, we aim to explore the cancer burden across districts in Oslo, and focus on the five most prevalent cancer types in Norway: colorectal, lung, melanoma, breast and prostate cancer [20]. These cancer types were also previously found to have varying incidence rates across education levels [21]. We aim to describe the distribution of cancer incidence and survival of these cancers in Oslo as a whole and in the administrative districts grouped according to level of education. We also present how the stage at time of diagnosis varies between the three education areas.

Health disparities within cities have been documented throughout Europe [22]. As such, the study’s relevance extends beyond its immediate context and can contribute to the broader international discussion on this topic, as well as the understanding of how health, disease and treatment are distributed along social divides.

## Method

### Data

Information on cancer diagnoses, date of diagnosis, and the patient’s place of residence was retrieved from the Cancer Registry of Norway. Diagnoses between 1 January 2013 and 31 December 2021 were included. This mainly involved patients who were 20 years and older, but a few individuals below this age (less than 10) were also included. The Cancer Registry of Norway has been considered complete since 1953, as clinicians and pathologists are required to report all cancer cases. The completeness was estimated to be 98.6% in 2018–2022 [23]. The registry provides basic information on patient and tumour characteristics as well as treatment. In recent years, it has also included more detailed information on diagnostic procedures and examinations, as well as follow-up for specific cancers [24].

The neoplasms included in this study have been classified according to the International Classification of Diseases, 10^th^ revision, and comprise colon (C18), rectum (C19–20), lung (C33-34), melanoma (C43), breast (C50), and prostate (C61).

### Districts and education areas

Oslo’s population is approximately 700,000 [25], and the city is organised into 15 administrative districts, each responsible for social, welfare, and healthcare services at the primary level. Data on the education level in each district in 2017 were obtained from Statistics Norway [26]. The Norwegian education system consists of basic school level and higher education. Basic education includes primary and secondary school, which spans a total of up to 13 years. Higher education options include college and university.

**Fig. 1:**
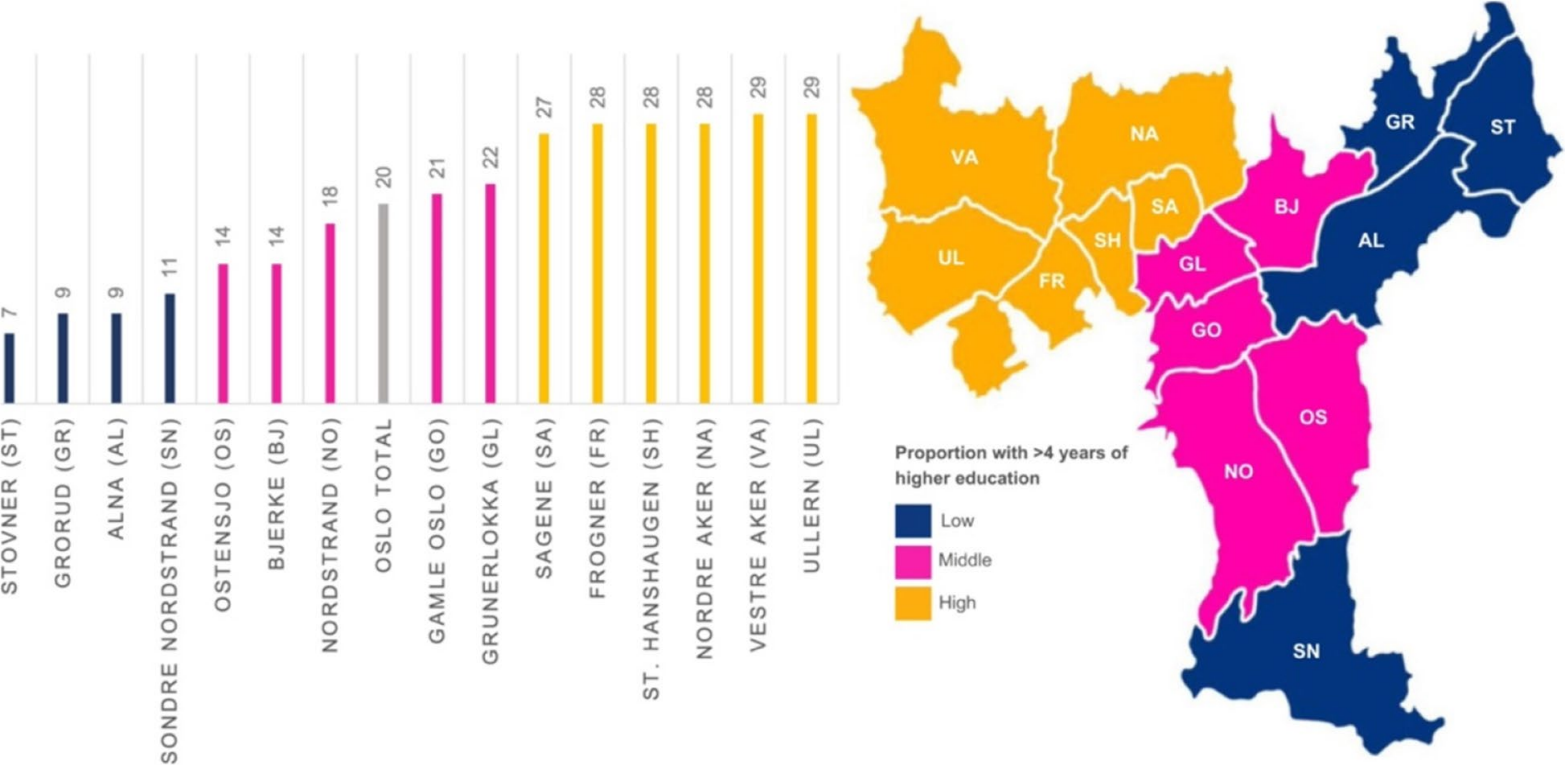
Percentage of residents aged ≥ 16 years with more than four years of higher education in 15 administrative districts of Oslo in the year 2017. (Oslo average shown in grey colour) [26]

The subgroups of the three education areas were based on the percentage of residents aged ≥ 16 years in each district who had completed more than four years of higher education. The education gaps between each group were large enough to make a natural categorization. The cut-off points were set at the 33^rd^ and 66^th^ percentile, with an intermediate group in between. We refer to these groups as low-, mid-, and high-education areas (Fig. 1).

Population characteristics in each administrative district are shown in Table 1. The data was obtained from Municipality of Oslo: District Facts [20]. In addition to having the lowest proportion with more than four years of higher education (6.6%–10.5%), the low-education area also had the highest proportion of residents with basic school level (range: 32.9%–38.7%), the highest proportion of immigrants (49.5%–55.4%) and unemployment rates (26.9%–30.2%), and the highest proportion of families with three or more children (4.2%–7.7%). In the mid-education area, some of the districts had an overweight of young people, in particular students. In the high-education area, between 26.0% and 28.8% of the residents had completed more than four years of higher education. Districts in this area had the highest median income and highest proportion of elderly residents, as well as the lowest proportion of immigrants and residents with basic school levels.

**Table 1:**
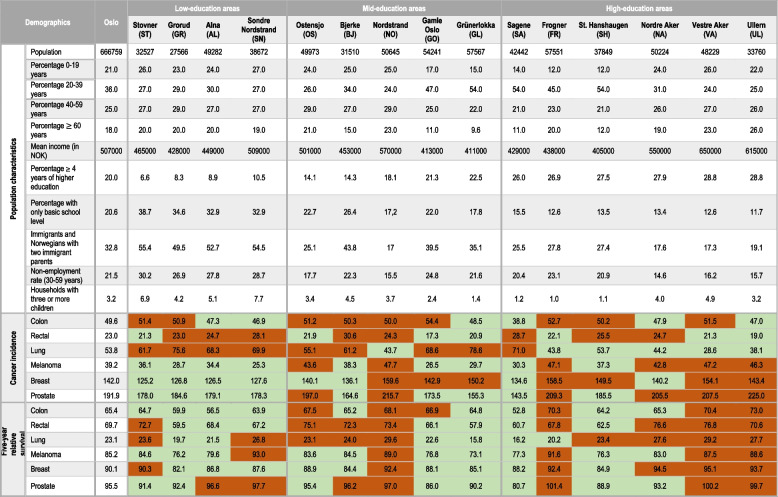
Demographics of the 15 administrative districts in Oslo in the year 2017 [25–27]. Age-standardised cancer incidence rates reported as cases per 100,000 person-years and five-year relative survival in the period 2013-2021. Red (darker) represents the districts with values equal to or higher than the Oslo average, whilst green (lighter) represents districts with values lower than the Oslo average

### Statistics

Age-standardised incidence rates were calculated by computing a weighted average of age-specific incidence rates using 18 age groups (0–4, 5–9, …, 85 +) and weights corresponding to the age distribution in the Norwegian population in 2014. The method is described in detail in Cancer in Norway Technical Supplement [28]. The age-standardised incidence rate was calculated for every district individually, as well as the three education areas, and is reported as cases per 100,000 person-years.

The stage at diagnosis was categorised into four groups after the Surveillance, Epidemiology, and End Results (SEER) summary staging system: localised: the cancer is confined to the primary site and has not metastasised to lymph nodes or other organs; regional: the tumour has invaded neighbouring tissue outside of the primary organ or metastasised to regional lymph nodes: distant: the tumour has metastasised to other organs or distant lymph nodes, and unknown: no information on stage, for all cancer types except breast cancer. The TNM system was used to stage breast cancer. This system is the combination of information on tumour size (T), regional nodes status (N), and metastases status (M) to categorise stages I-IV and unknown. Staging of breast cancer is complex. In short, stage I is breast cancer in an early stage whereas the tumour has distant metastases in stage IV. The stage at diagnosis was calculated for the period 2013–2021.

Five-year relative survival was estimated using the Pohar-Perme (PP) estimator with individual weights for age [29]. The PP estimator calculates net survival, i.e., the probability of surviving cancer when cancer is the only possible cause of death, by weighting each individual with the inverse of their expected survival probability, The weights inflate the observed person-time and number of deaths to account for person-time and deaths not observed as a result of mortality due to competing causes. Consequently, the survival experience of older patients gets upweighted relative to the survival experience of younger patients. The weights used for age-standardisation were based on the cancer patients’ age distribution in 2017–2021. The national lifetable used in the estimation was stratified by sex, one-year age groups and calendar year. For more detail on this method, see Cancer in Norway Technical Supplement [28]. Complete vital information was available until 31 December 2022. All statistical analyses were performed using Stata version 17.0 [30].

## Results

Our study included information on 15,830 cancer cases diagnosed between 2013 and 2021 among patients living in Oslo. In total, there were 2372 cases of colon cancer cases, 1115 rectal cancer, 2540 lung cancer, 1981 melanoma, 3729 breast cancer, and 4093 prostate cancer. 23.3% of the patients were in the low-education area, 29.0% in the mid-education area, and 47.8% in the high-education area.

### Incidence

#### Districts

Table 1 displays age-standardised cancer incidence rates across the 15 districts. In the low-education area, none of the districts had higher incidence rates than the Oslo average for melanoma, breast, and prostate cancer. However, compared to the Oslo average, all districts in this area had a higher incidence rate for lung cancer, and three out of four districts had a higher incidence rate for rectal cancer. Half of the districts had higher incidence rates for colon cancer than the Oslo average.

In the high-education area, most districts have a higher incidence rate for melanoma, breast, and prostate cancer than the Oslo average. All districts in this area, except one, have lower incidence rates for lung cancer. Half of the districts have higher rates of colon and rectal cancer than Oslo.

#### Areas

**Fig. 2:**
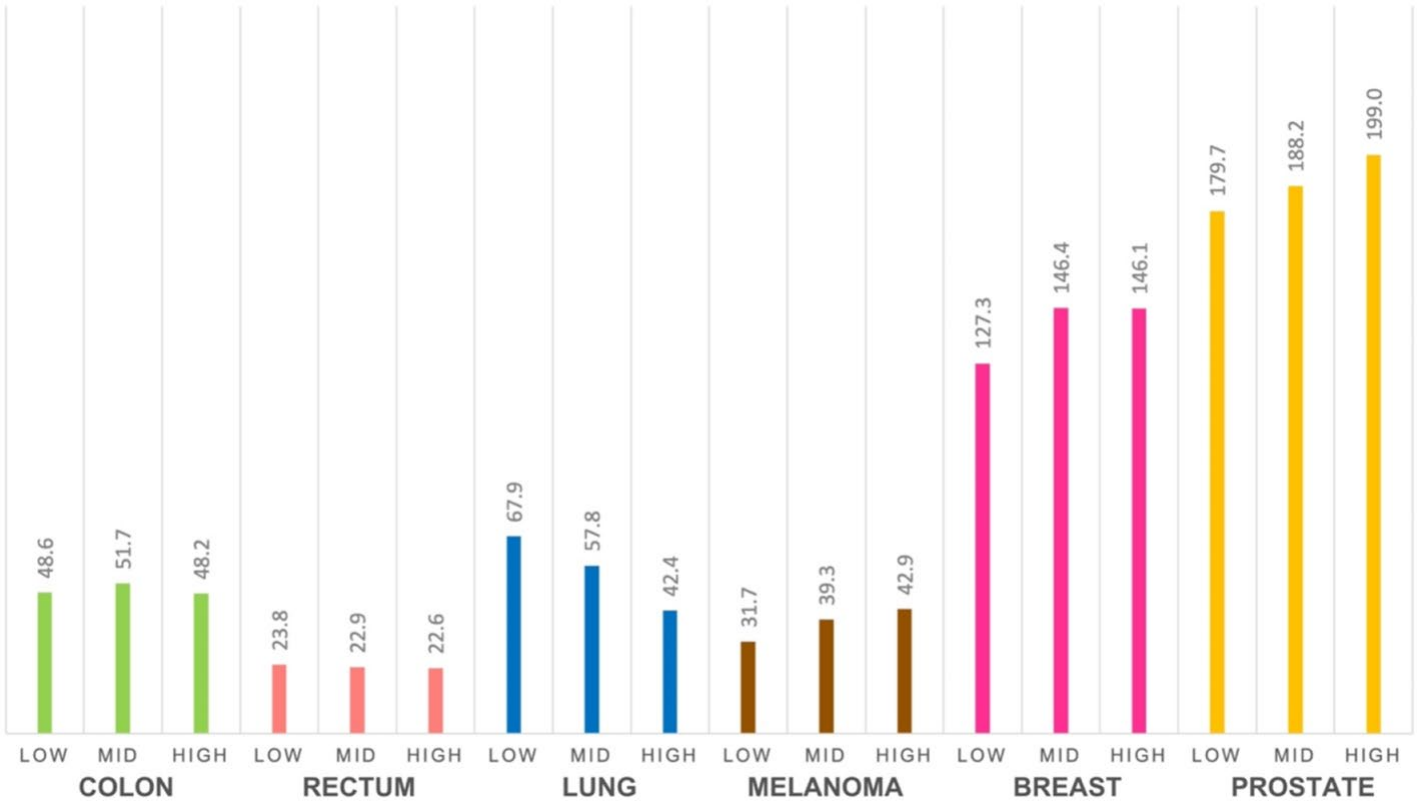
Age-standardised incidence rates (cases/100,000 person-years) for six cancer types diagnosed in 2013–2021 among residents in three areas of Oslo, categorised by low-, mid-, and high-education levels

**Fig. 3:**
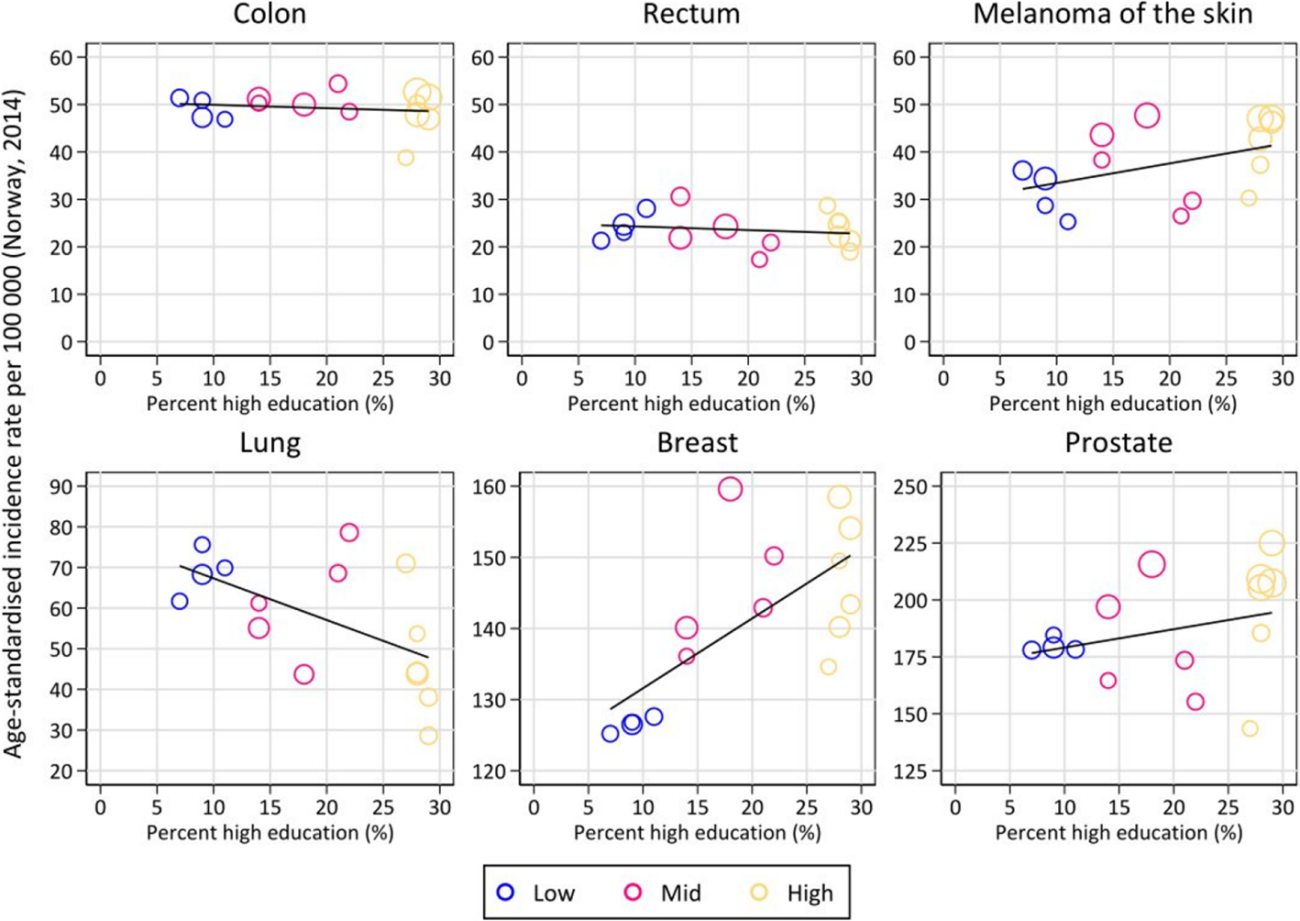
Association between age-standardised incidence rates reported as cases per 100,000 person-years and percentage with more than four years of higher education for the six cancer types in each district. The districts are categorised into low-, mid-, and high-education areas by colour. Black line represents the linear association between the two measures

Figures 2 and 3 display cancer incidences across the three education areas. There was a higher incidence with higher educational level for prostate cancer and melanoma (90.7 cases/100,000 person-years in the high-education area compared to 81.8 in the low for prostate cancer, and 42.9 in the high-education area compared to 31.7 in the low for melanoma). Regarding breast cancer, the mid-, and high-education areas had a higher incidence compared to those in low-education areas (76.0 and 76.1 compared to 67.1). For lung cancer, there was lower incidence with an increasing level of education (67.9 compared to 42.4). The incidence rates for colon and rectal cancer were evenly distributed across low-, mid-, and high-education areas (Figs. 2 and 3).

### The stage at time of diagnosis across areas

The percentage of colon cancer and melanoma diagnosed at a localised stage was higher in the high-education area (respectively, 17.0%, and 83.8% in the high-education area, compared to 14.0% and 79.8% in the low). Further, the high-education area never had the highest proportion of cancer diagnosed at a distant stage (Table 2).

**Table 2:** Percentage of cancer cases at different stages at the time of diagnosis. Categorised in the low-, mid-, and high-education areas of Oslo, from 2013 to 2021 (Range in parentheses)

		Education area		
**Colon cancer**		**Low (*n* = 541)**	**Mid (*n* = 717)**	**High (*n* = 1114)**
**Stage**	Localised	14.0 (9.6–17.5)	17.4 (14.7–19.1)	17.0 (11.6–21.6)
	Regional	50.4 (43.5–59.3)	49.2 (45.3–53.3)	50.2 (44.8–52.9)
	Distant	26.0 (23–29.6)	21.6 (16.1–31.6)	25.2 (21.1–33.9)
	Unknown	9.4 (7–12)	11.8 (5.1–14.7)	7.6 (5.0–14.6)
**Rectal cancer**		**Low (*n* = 273)**	**Mid (*n* = 322)**	**High (*n* = 520)**
**Stage**	Localised	24.6 (20.3–29.4)	21.4 (16.9–26.1)	23.4 (15.1–29.7)
	Regional	45.0 (34.3–55.9)	44.8 (30.4–53.8)	43.0 (34.7–50.0)
	Distant	22.4 (18.6–29.9)	19.6 (13.8–24.6)	20.4 (15.5–26.4)
	Unknown	8.0 (5.1–11.9)	14.2 (11.4–19.6)	13.0 (9.4–19.0)
**Lung cancer**		**Low (*n* = 770)**	**Mid (*n* = 725)**	**High (*n* = 1045)**
**Stage**	Localised	20.6 (17.5–22.1)	25.0 (23.7–26.5)	20.0 (16.3–25.3)
	Regional	26.2 (23.5–29.5)	26.4 (24.4–27.8)	26.6 (20.5–31.9)
	Distant	43.8 (35.8–50.0)	32.2 (26.1–41.0)	38.2 (29.9–46.9)
	Unknown	9.4 (6.3–12.7)	16.6 (9.6–21.4)	15.2 (7.1–24.0)
**Melanoma**		**Low (*n* = 367)**	**Mid (*n* = 585)**	**High (*n* = 1029)**
**Stage**	Localised	79.8 (76.6–83.1)	83.2 (76.4–91.8)	83.8 (74.0–88.2)
	Regional	9.6 (6.0–10.9)	8.2 (4.7–11.2)	8.2 (5.5–14.3)
	Distant	4.0 (1.5–7.5)	3.6 (3.6–4.7)	4.0 (3.2–7.8)
	Unknown	6.6 (1.5–10.9)	5.0 (3.5–7.9)	4.0 (2.0–5.7)
**Breast cancer**		**Low (*n* = 797)**	**Mid (*n* = 1106)**	**High (*n* = 1826)**
**Stage**	Stage I	43.6 (40.5–48.6)	40.4 (37.9–44.2)	43.4 (38.2–50.3)
	Stage II	30.8 (23.7–33.8)	33.0 (30.3–34.4)	29.4 (24.9–35.0)
	Stage III	11.2 (8.9–14.4)	11.0 (8.3–13.6)	12.4 (9.9–16.2)
	Stage IV	2.8 (0.6–4.5)	5.0 (4.3–5.9)	5.0 (2.0–8.4)
	Unknown	11.6 (8.7–14.6)	10.6 (8.3–16.0)	9.8 (8.3–11.4)
**Prostate cancer**		**Low (*n* = 935)**	**Mid (*n* = 1131)**	**High (*n* = 2027)**
**Stage**	Localised	45.6 (43.7–49.3)	41.4 (36.0–42.9)	42.0 (29.9–45.3)
	Regional	31.0 (36.0–42.9)	35.8 (34.3–37.3)	34.0 (29.7–42.5)
	Distant	9.0 (7.5–11.5)	7.4 (5.9–8.9)	5.8 (4.1–11.0)
	Unknown	14.4 (10.6–16.1)	15.2 (14.2–19.3)	18.2 (14.4–20.3)

Rectal, breast and prostate cancer diagnosed at a localised stage were more frequent in the low-education area (respectively, 24.6%, 43.6% and 45.6% in the low-education area, compared to 23.4, 43.4 and 42.0% in the high). However, this area had the highest proportion of cancer diagnosed at a distant stage for all cancer types except breast cancer.

### Survival

#### Districts

Table 1 displays relative survival across the 15 districts. Three out of four districts in the low-education area had lower cancer survival than the Oslo average for all cancer types, except for lung and prostate cancer where two out of four districts had lower survival than the Oslo average.

In the high education area, most districts had higher survival than Oslo for rectal, lung, and breast cancer. For colon, melanoma, and prostate cancer, the area was split in half, with one-half of the districts having a higher relative survival than the Oslo average.

#### Areas

**Fig. 4:**
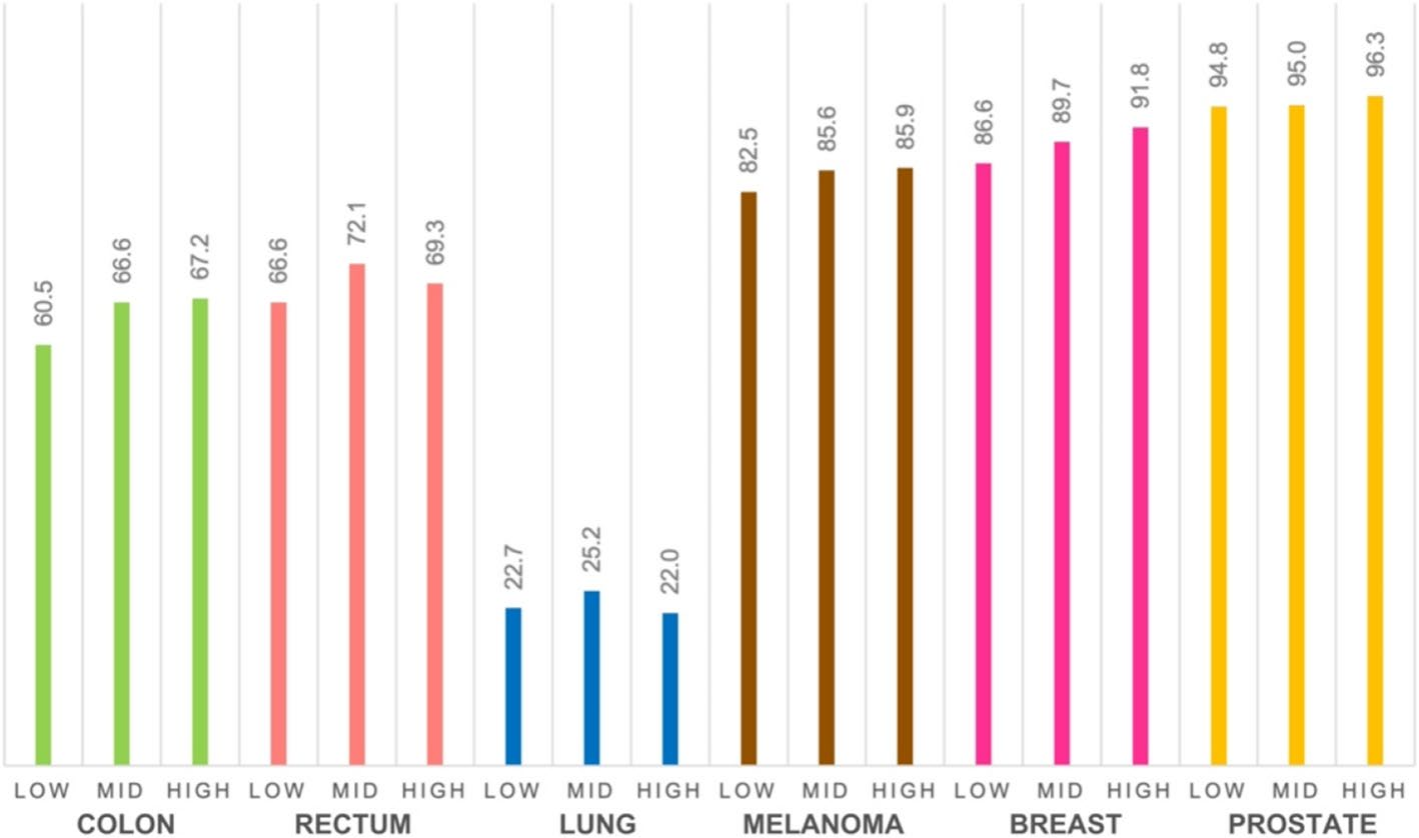
Five-year relative survival for six cancer types diagnosed in 2013–2021 among residents in three areas of Oslo, categorised by low-, mid-, and high-education levels

Figure 4 displays the five-year relative survival across the three education areas. Five-year relative survival was higher in mid- and high-education areas for all six cancer types. Relative survival was highest in the high-education area for colon, melanoma, breast, and prostate cancer, and highest in the mid-education area for rectal and lung cancer. The low-education area had the lowest relative survival for all cancer types except lung cancer, whereas the high-education area had the lowest relative survival (Fig. 4). One-year relative survival estimates are presented in Online Resource 2.

## Discussion

The results of this study suggest that there are small, yet notable, differences in cancer incidence, stage at the time of diagnosis, and survival across districts with differences in educational levels in Oslo, Norway.

Based on previous studies, we assumed that there would be a higher incidence of lung and rectal cancer in the low-education area [5, 31], while the high-education area would have higher incidences of breast [5, 10, 32, 33], prostate [5, 34] and melanoma cancer [5, 35]. For colon cancer, we assumed there would be an even distribution across the education areas [5]. The main findings in our study were in coherence with previous studies; a higher incidence of lung cancer was found in the low-education area, while melanoma, prostate and breast cancer had higher incidence rates in the high-education area. However, there was an even distribution of both colon and rectal cancer incidence across the education areas.

We further assumed that in areas with a higher educated population, more cancer cases would be diagnosed at an earlier stage [36, 37]. We found that in the low-education area, all cancer types, except breast cancer had the highest proportion diagnosed at a distant stage.

Finally, we assumed that areas with a higher educated population would have higher survival [38–42] for all cancer types. With the exception of lung cancer, this assumption was also confirmed, the low-education area had the lowest five-year relative survival for the other five cancer types. Although the high-education area had the highest incidence for most cancer types, relative survival was also higher for the same types. This is an intriguing observation that merits further investigation.

Better survival might be the result of better access to and utilization of healthcare and cancer screenings, perhaps resulting in increased consultations [43]. Income, patient support network, and health literacy (the ability to understand and navigate the healthcare system), contribute to better access, for example, through easier communication with the system, or the ability to afford private health services. Further, studies in other fields have shown that educational level plays a role in the choice of treatment [44], so it is important to explore the possibility that educational level influences the choice of cancer treatment. District affiliation alone does not decide the place of treatment for specialist healthcare in Oslo. It is determined by a combination of factors, the most important being the availability of specialized medical facilities, the specific medical condition or treatment needed, the recommendations of referring physicians, and the patient’s own choice.

Breast, prostate, lung cancer and melanoma were among the cancer types that we expected to be influenced by education. With lung cancer, smoking is a well-known risk factor [45], and studies show an association between smoking behaviour and educational level, with higher rates of smoking among lower education individuals [46–48]. For breast cancer, increased risk has been linked to not having children and late childbirth [49, 50], which tend to be more prevalent among women with higher levels of education who might delay childbearing to complete longer education [10, 51, 52]. In Norway, there are national screening programs for breast cancer, and individuals with higher education are more likely to participate [53]. Unlike breast cancer, there are no organized screening programs for prostate cancer or melanoma, hence individuals need to be proactive in requesting tests. Health literacy plays a role in this regard.

The results of our study are in line with other studies on the social distribution of health and illness. To our knowledge, this is the first study of cancer incidence, stage, and survival in Oslo, thus contributing to more knowledge about social disparities in this city.

### Limitations and strengths

Limitations include the lack of data on comorbidities, which may influence relative survival. As with any observational study, it is not possible to establish causality between education level and cancer outcome. We could also not account for individual factors as we used aggregated data.

However, using data from the Cancer Registry of Norway ensured that we had complete cancer data for the entire population of Oslo over nine years. In addition to the high-quality dataset, this study had several other strengths. Studies are often conducted on a larger scale, with national or international focus. By focusing on a smaller scale, we aimed to gain a better understanding of the specific issues and challenges in Oslo. We were able to reduce the influence of some of the factors associated with cancer incidence, stage at diagnosis and survival. Such factors might be accessibility and availability to general practitioners, specialist health care services and resources, distance to health services and environmental factors. The different districts of Oslo are relatively similar in terms of access to primary care, as well as the distance to hospitals only being a couple of kilometres. Further, the districts are relatively similar regarding environmental exposures, and access to green spaces, and there are no districts with extreme poverty or slums.

### Implications and interpretations

This study provides insight into the disparities in cancer incidence, stage at the time of diagnosis, and survival rates across districts with differences in education levels in Oslo, Norway.

Although the incidence rates for some cancer types were higher in the high-education group, the survival was lower for all cancer types, except lung, in the low-education group. Further, for four out of six cancer types, the low-education area had the highest proportion diagnosed at the distant stage.

Our findings can inform targeted prevention, diagnostic, and treatment efforts in Oslo as it allows for a more targeted and localized approach to policymaking. By pinpointing the areas where disparities exist, we can tailor interventions to effectively target and improve the health outcomes of specific communities. Studying a diverse city like Oslo provides insights that can inform and guide efforts to reduce health inequities in other urban areas.

We chose education as the distinguishing criterion between socio-economic groups because health literacy is shown to play an important role in health outcomes[54, 55]. Our study supports this. Having a good understanding of health literacy helps you identify potential warning signs, ask important questions, and make informed decisions about your health.

Additionally, our findings highlight the need for more research on the mechanisms behind these disparities and the efficacy of interventions to reduce them. For instance, studies could investigate the correlation between educational level and health behaviours or assess whether differences in patient-doctor communication and choice of treatment contribute to the observed disparities. Such studies would add to the relatively scant empirical knowledge about mechanisms behind social inequalities in the healthcare system. This is a necessary step in order to develop policies aiming to reduce inequities.

Overall, our study contributes to the broader international discussion on health disparities within cities and the importance of addressing the social determinants of health.

### Supplementary Information

**Additional file 1: Online Resource 1.** One-year survival rates 2013-2021 in the low-, mid-, and high-education areas, as well as the Oslo average. (Range in parentheses).

**Additional file 2: Online resource 2.** Five-year relative survival for the six selected cancers in the low-, mid-, and high-education areas, as well as the Oslo average. (Range in parentheses).

## Data Availability

The data that support the findings of this study are publicly available upon request.
