# Supplementary material for: Educational disparities in cancer incidence, stage, and survival in Oslo
Source: Res Health Serv Reg. 2024 Jan 29;3:1. doi: 10.1007/s43999-024-00037-x (PMC11281764; doi:10.1007/s43999-024-00037-x)
Supplement: Supplementary file 1 — Additional file 1: Online Resource 1. One-year survival rates 2013-2021 in the low-, mid-, and high-education areas, as well as the Oslo average. (Range in parentheses). [file 43999_2024_37_MOESM1_ESM.pdf]

Online Resource 1 One-year survival rates 2013-2021 in districts with a lower, middle, and higher educated population, as well as Oslo overall

	Low	Middle	High	Oslo
**Colon**	81.5 (78.9, 84.2)	84.6 (82.4, 86.8)	83.5 (81.6, 85.4)	83.3 (82.1, 84.6)
**Rectal**	85.9 (82.4, 89.5)	86.4 (83.5, 89.4)	87.6 (85.2, 90.1)	87.0 (85.4, 88.6)
**Lung**	47.7 (44.8, 50.8)	51.1 (48.2, 54.1)	49.8 (47.3, 52.3)	49.5 (47.9, 51.1)
**Melanoma**	93.5 (91.0, 96.2)	94.2 (92.1, 96.2)	95.7 (94.2, 97.2)	94.8 (93.7, 95.9)
**Breast**	96.2 (94.6, 97.8)	97.6 (96.4, 98.7)	97.8 (96.9. 98.7)	97.4 (96.7, 98.0)
**Prostate**	99.4 (98.4, 100.4)	98.7 (97.8, 99.7)	99.4 (98.7, 100.1)	99.2 (98.7, 99.7)

TITLE: Educational Disparities in Cancer Incidence, Stage at Diagnosis and Survival in Oslo

JOURNAL: Research in Health Services and Regions

AUTHORS AND AFFILIATIONS

Afaf Al-Rammahy [a, b], Elin Anita Fadum [b, d], Yngvar Nilssen [c], Inger Kristin Larsen [c], Erlend Hem [a, b], Berit Horn Bringedal [b]

[a] Department of Behavioural Medicine, Institute of Basic Medical Sciences, Faculty of Medicine, University of Oslo, Norway.

[b] Institute for Studies of the Medical Profession, Oslo, Norway.

[c] Department of Registration, Cancer Registry of Norway, Oslo, Norway

[d] The Norwegian Armed Forces Joint Medical Services, Institute for Military Epidemiology, Sessvollmoen N-2018, Norway

CORRESPONDING AUTHOR

Afaf Al-Rammahy, afafa@uio.no
