# Supplementary material for: Educational disparities in cancer incidence, stage, and survival in Oslo
Source: Res Health Serv Reg. 2024 Jan 29;3:1. doi: 10.1007/s43999-024-00037-x (PMC11281764; doi:10.1007/s43999-024-00037-x)
Supplement: Supplementary file 2 — Additional file 2: Online resource 2. Five-year relative survival for the six selected cancers in the low-, mid-, and high-education areas, as well as the Oslo average. (Range in parentheses). [file 43999_2024_37_MOESM2_ESM.pdf]

Online resource 2 Five-year relative survival 2013–2021 for the six selected cancers in the low-, mid-, and high-education areas, as well as the Oslo average. (Range in parentheses)

	Oslo	Low	Mid	High
**Colon**	65.4 (63.4, 67.4)	60.5 (56.6, 64.7)	66.6 (63.1, 70.2)	67.2 (64.4, 70.2)
**Rectal**	69.7 (67.0, 72.5)	66.6 (61.2, 72.6)	72.1 (67.4, 77.1)	69.3 (65.3, 73.6)
**Lung**	23.1 (21.7, 24.7)	22.7 (20.1, 25.7)	25.2 (22.5, 28.2)	22.0 (19.8, 24.5)
**Melanoma**	85.2 (82.9, 87.5)	82.5 (77.5, 87.8)	85.6 (81.7, 89.7)	85.9 (82.6, 89.3)
**Breast**	90.1 (88.6, 91.7)	86.6 (83.3, 90.1)	89.7 (86.8, 92.6)	91.8 (89.7, 94.0)
**Prostate**	95.5 (94.2, 96.9)	94.8 (92.0, 97.8)	95.0 (92.4, 97.5)	96.3 (94.4, 98.3)

TITLE: Educational disparities in cancer incidence, stage at diagnosis and survival in Oslo

JOURNAL: Research in Health Services and Regions

AUTHORS AND AFFILIATIONS

Afaf Al-Rammahy [a, b], Elin Anita Fadum [b, d], Yngvar Nilssen [c], Inger Kristin Larsen [c], Erlend Hem [a, b], Berit Horn Bringedal [b]

[a] Department of Behavioural Medicine, Institute of Basic Medical Sciences, Faculty of Medicine, University of Oslo, Oslo, Norway.

[b] Institute for Studies of the Medical Profession, Oslo, Norway.

[c] Department of Registration, Cancer Registry of Norway, Oslo, Norway

[d] The Norwegian Armed Forces Joint Medical Services, Institute for Military Epidemiology, Sessvollmoen N-2018, Norway

CORRESPONDING AUTHOR

Afaf Al-Rammahy, afafa@uio.no
